# Evaluation of time in therapeutic range in anticoagulated patients: a single-center, retrospective, observational study

**DOI:** 10.1186/1756-0500-7-891

**Published:** 2014-12-09

**Authors:** Daniel Caldeira, Inês Cruz, Gonçalo Morgado, Bruno Stuart, Catarina Gomes, Cristina Martins, Isabel João, Hélder Pereira

**Affiliations:** Cardiology Department, Hospital Garcia de Orta, Serviço de Cardiologia, Av.Torrado da Silva, 2805-267 Almada, Portugal; Clinical Pharmacology Unit, Instituto de Medicina Molecular, Lisbon, Portugal; Laboratory of Clinical Pharmacology and Therapeutics, Faculty of Medicine, University of Lisbon, 1649-028 Lisbon, Portugal

**Keywords:** Anticoagulation quality, TTR, Warfarin, Vitamin K antagonist

## Abstract

**Background:**

The percentage of time during which the patients have the INR within the target values (i.e. Time in Therapeutic Range [TTR]) is a measure of anticoagulation quality with Vitamin K Antagonists (VKA). To evaluate the quality of anticoagulation using TTR according to the Rosendaal method, we performed an observational, retrospective study. We included all outpatients who attended the cardiology anticoagulation clinic of a Portuguese hospital (2011–2013), whose target INR was 2.0-3.0.

**Results:**

377 VKA-treated patients were evaluated. Of these, 72.4% had non-valvular atrial fibrillation. Patients were followed for a mean period of 471 days. The mean TTR was 60.3% (SD 19.3%) and 44.3% of the patients had a mean TTR < 60%. Patients were at high risk of bleeding (INR > 4.5) and at high thrombotic risk (INR < 1.5) during, respectively, 1.7% and 4.7% of the time.

**Conclusions:**

Anticoagulation control needs to be improved. These results are informative for all stakeholders: patients, health care professionals, and policymakers.

## Background

Vitamin K Antagonists (VKA) such as warfarin, acenocoumarol and phenpromcom are widely prescribed oral anticoagulant drugs. The main indications are atrial fibrillation (AF), valvular prosthesis, venous thromboembolism and intracavitary thrombus. These drugs’ efficacy and safety depends on International Normalized Ratio (INR) monitoring. The absence of standard dosages of VKA turns imperative to perform serial INR tests and make dosages adjustments when the results are out of the range.

INR levels above and under pretended values are associated to increased risk of hemorrhagic and thromboembolic events, respectively [1, 2].

Time in therapeutic range (TTR) is a measure of quality of anticoagulation and lower values are related to adverse events [3].

TTR knowledge is important to identify the current standard of anticoagulation care and establish new goals. Additionally TTR is an important input to determine the cost-effectiveness of new oral anticoagulants [4].

The most comprehensive published data about TTR in Portuguese patients comes from RE-LY study. This trial included Portuguese patients and mean TTR was 61% [5, 6].

TTR data retrieved from randomized controlled trials may overestimate those from real world [7]. Therefore we aimed to retrospectively review the charts of patients from a single-center anticoagulation consultation in order to estimate TTR.

## Methods

### Study design and setting

We conducted a retrospective cohort study of patients treated with vitamin K antagonists followed in Cardiology Anticoagulation Clinic a Portuguese single-centre from January 2011 to July 2013, in order to determine the TTR of the centre. We obtained Institutional Board and Ethics Committee approval for this study.

### Participants, variables and statistical analysis

We identified all patients treated with vitamin K antagonists followed in the Outpatient Cardiology Anticoagulation Clinic. Patients’ data were retrieved from a database which contains the all INR records obtained in the visits. All patients were submitted to nurse led INR checking using CoaguCheck® XS system and follow-up was made according to INR value, and hospital protocol or physicians preferences.

For analysis, we included patients whose target INR was between 2.0 and 3.0 (patients with INR targets between 2.5 to 3.5, including patients with mechanical heart valves were excluded). To better characterize the quality of long-term anticoagulation all patients under 2 months of follow-up tests or <6 INR tests were excluded [8]. We have characterized the demographic and clinical characteristics of the population. For each patient we evaluated all available INR values to calculate the individual TTR according to the Rosendaal method [9]. This method uses linear interpolation to assign an INR value to each day between successive observed INR values.

Patients were clustered into subgroups according to the reason/indication for anticoagulation: non-valvular AF; valvular AF (patients with mitral stenosis, severe aortic stenosis, severe mitral regurgitation, biologic prosthetic valves, valve repair); venous thromboembolic disease; and others (including left ventricular dysfunction, intracavitary thrombus, non-compaction cardiomyopathy).

The primary outcome was the TTR, a continuous outcome. Secondary outcomes were: 1) TTR < 60%, a marker of poor quality in the control of INR [3, 10]; 2) time under therapeutic range (INR < 2.0); 3) time over therapeutic range (INR > 3.0); 4) time with increased thrombotic risk (INR < 1.5); 5) time with increased hemorrhagic risk (INR > 4.5).

All analyses were conducted using SPSS software version 9.1. Statistical summary measures such as arithmetic mean and median were used to characterize the population. Standard deviation (SD) and interquartile range were used to evaluate data dispersion. Multivariate logistic regression analysis was performed to identify risk factors for TTR < 60%, at a significance level of 0.05. Chi-square test was performed for the comparison of dichotomic data across groups. One-way ANOVA was used to evaluate differences between TTR across indications (more than 2 groups). The results were considered to be statistically significant at a p-value <0.05.

### Ethics

Hospital Garcia de Orta Institutional Board and Ethics Committee have approved this project.

## Results

We found 501 patients treated with VKA with target INRs between 2.0 and 3.0, with their INR recorded in the database between January 2011 and July 2013. About 377 patients had the minimum required follow-up/number of tests to meet the inclusion criteria.

The mean age was 71 years, and 59.4% of the patients were male. Most of the patients had non-valvular AF (72.4%), while valvular AF (19.1%) and venous thromboembolic disease (3.4%) were less common. The population’s average CHA_2_DS_2_-VASc was 3.58.

Patients were followed for a mean period of 471 days, having performed on average 17 INR tests per year each patient. The average time between two tests was 25.4 days.

Table 1 shows the main characteristic of the population.

**Table 1:** **Main characteristic of included patients**

Characteristics	Population (N = 377)
Age – years	
Mean (SD)	71.0 (10.4)
Median (IQR)	72.0 (66–79)
Female sex – no. (%)	153 (40.6)
Previous stroke or transient ischemic attack – no. (%)	56 (14.9)
Heart failure – no. (%)	160 (42.4)
Diabetes mellitus – no. (%)	101 (26.8)
Hypertension - no. (%)	253 (67.1)
Vascular Disease History – no. (%)	122 (32.4)
Indication for anticoagulation	
Non-valvular AF	273 (72.4)
Valvular AF*	72 (19.1)
Venous thromboembolism	13 (3.4)
Others	19 (5.1)
CHA_2_DS_2_-VASc	
Mean (SD)	3.58 (1.62)
Median (IQR)	3 (2–5)

*Mitral stenosis, severe aortic stenosis, severe mitral regurgitation, biologic prosthetic valves, valve repair. AF: Atrial Fibrillation; IQR: Interquartile Range; SD: Standard Deviation

The mean TTR was 60.3% (SD 19.3%) and the median 63% (interquartile range 47.9-74.8%). About 44.3% of the patients evaluated have a mean TTR < 60%, and are at increased risk of thrombotic and hemorrhagic events.

The female gender was the only characteristic that was significantly associated to poor anticoagulation control (TTR < 60%) in the multivariable regression analysis with an odds ratio 1.73 and 95% confidence interval 1.14-2.62 (p = 0.01).

**Figure 1:**
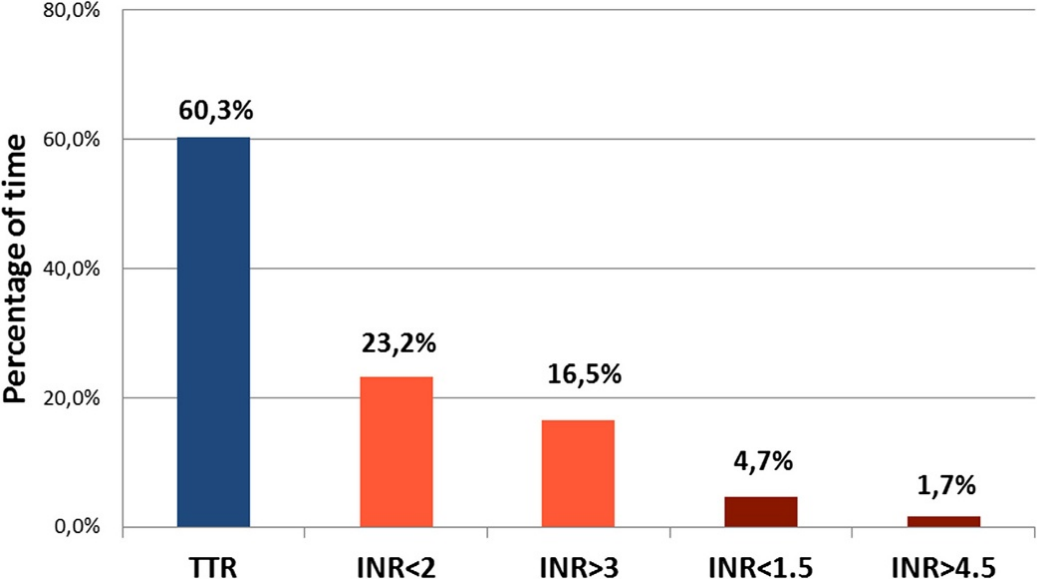
**Time in therapeutic range (TTR) and time out of therapeutic range.**

**Figure 2:**
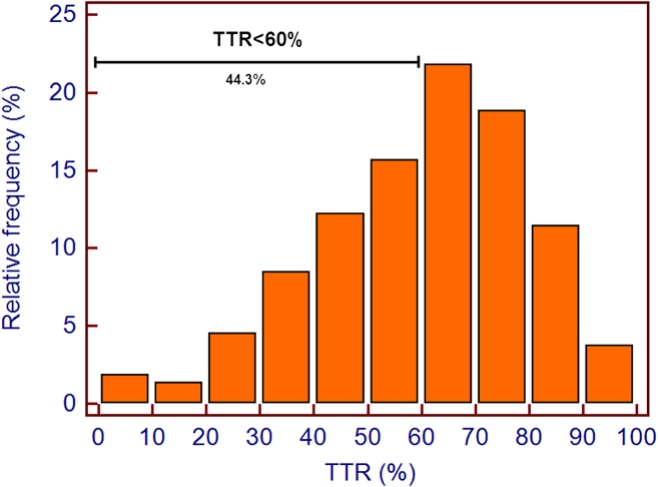
**Histogram with relative frequencies of TTR values and proportion of patients with TTR < 60%.**

The average percentage of time that patients remained above (INR > 3.0) and below the target INR (INR < 2.0) was 16.5% and 23.2%, respectively. Patients were at high risk of bleeding (INR > 4.5) 1.7% of the time, and at high thrombotic risk (INR < 1.5) 4.7% of the follow-up period. Figures 1 and 2 illustrate these results.

Non-valvular AF was the most prevalent indication for anticoagulation. The mean CHA_2_DS_2_-VASc was 3.65 (SD 1.58). In this cluster of patients, the average TTR was 59.3% (SD 19.8%) and the median was 61.8% (interquartile range 47.4-73.7%). These patients were on average 23.4% of the time below therapeutic range (INR < 2.0), and 17.3% of the time over INR 3.0. The mean percentage of time with high thrombotic risk (INR < 1.5) was 5.3%, and 1.7% of the time patients were at high risk of bleeding.

There were no significant differences in average TTR between the different indications for VKA treatment (p = 0.18). The proportion of patients with low anticoagulation control also was not different across conditions (p = 0.53). Table 2 shows the mean TTR and the proportion of TTR < 60% according to the main indication for anticoagulation.

**Table 2:** **Mean Time in Therapeutic Range (TTR) according to main indication for anticoagulation**

Population	Mean TTR (SD)	TTR < 60% (%)	Patients	Average follow-up (years)
Non-valvular AF	59.3% (19.7%)	128 (46.7%)	274	1.27
Valvular AF	64.0% (18.6%)	29 (40.3%)	72	1.44
Venous thromboembolism	54.6% (24.4%)	5 (38.5%)	13	1.18

AF: Atrial Fibrillation; SD: Standard Deviation; TTR: Time In Therapeutic Range.

## Discussion

VKA have been shown to be effective in the treatment and prevention of thromboembolic events, however they possess many drug-drug and drug-food interactions, as well as a narrow therapeutic window. Despite high number of studies in the field, much of the individual variability in response to warfarin therapy remains unexplained and, therefore careful monitoring is required in order to reduce the risk of tromboembolic events and bleeding complications. This process is costly and inconvenient for many patients [11]. The quantification of TTR allows characterization and of anticoagulation control quality.

According to our study, the TTR of this population of anticoagulated Portuguese patients was 60.3% during a mean follow-up of 1.3 years. Forty-four percent of this population had a TTR < 60%. This means that an important proportion of patients are at increased risk of major adverse events [3, 10, 12].

The results show an inadequate control of anticoagulation from a global point of view [13, 14]. Additionally, the identification of the female gender as a predictor of low TTR goes in line with the recent SAMe-TT_2_R _2_ that identifies women (Sex – female; S in the acronym) as population at risk for inadequate anticoagulation with VKA [15].

Recent large trials with new oral anticoagulants in AF provided further data about world-wide quality of anticoagulation control. In ROCKET-AF the mean TTR was 55.2% (63% in Western Europe, 64% in North America) [16], and the median TTR was 66% in ARISTOTLE [17]. The RE-LY study had a median TTR of 67.2% and presented TTR data according to countries, including Portugal. The benchmark countries were Sweden, Finland, and Australia with TTR values of 77% (Sweden) and 74% (Finland and Australia) [6]. The results obtained in our study are similar to those of the RE-LY study for Portugal (61%), and are overall in accordance to those reported in the literature [7, 18].

In our study we calculated individual patient TTR using a longitudinal linear extrapolation of INR values through a method proposed by Rosendaal as it is more time sensitive than other methods (takes into account the number of days within the range) [19].

The experience of other national centres about the quality of anticoagulation control reports data of INR tests within pretended ranges, rather than longitudinal TTR method. In an anticoagulation clinic 1067 INR controls were performed in two months in 687 patients. About 71% of the tests were within the range [20]. Another single centre experience of INR telemonitoring showed that 83% of the tests were within the range [21].

Applying our data to those retrieved from randomized controlled trial, in centres with TTR of 61%, new oral anticoagulants tend to be safer and/or more effective than VKA. In RE-LY, all dosages (110 mg and 150 mg bid) had a significant lower risk of intracranial bleeding, with a similar risk major bleeding, while in the prevention of thromboembolic events only the dosage of 150 mg showed a significant risk reduction compared to warfarin. The efficacy of rivaroxaban was not statistically different from warfarin but there was a trend towards rivaroxaban in the prevention of thromboembolic complications (HR 0.70; IC95% 0.48-1.03). Apixaban was safer in terms of major bleeding with an efficacy likely to be better than warfarin (HR 0.73; IC95% 0.53-1.00).

### Limitations

The main limitation of this study was that it was a retrospective, non-randomised cohort of patients anticoagulated with VKA followed in the Cardiology Anticoagulation Consultation of a single-center. However this is still, to the best of our knowledge, the first study evaluating the quality of anticoagulation in Portugal with Rosendaal TTR.

Patients with non-valvular AF with stable therapeutic INR values are usually proposed for discharge to primary care follow-up. The data here presented does not account for INR values registered in other facilities, such as in the emergency room or during hospitalizations. These reasons may limit the conclusions of this study.

We did not focus on other patients with very high thrombotic risk such as those carrying mechanical heart valves (because INR target is 2.5-3.5). So the data here presented cannot be extrapolated to such subgroups.

We used CHA_2_DS_2_-VASc score all patients, nevertheless we recognize that the use of such tool in valvular AF or in VTE may not be adequate. This score identifies prevalent risk factors for thromboembolism and we used to describe the population without performing any analysis on this basis.

## Conclusions

The average TTR of this center was 60.3%. An important proportion of patients was at high risk of events (TTR < 60%). At our center, anticoagulation control should be improved. When out of therapeutic range, patients were more commonly prone to prothrombotic risk due to the higher percentage of time with INR < 2.0. These results are informative for all stakeholders: patients, health care professionals, and policymakers.

## Abbreviations

AF: Atrial fibrillation
INR: International normalized ratio
TTR: Time in therapeutic range
VKA: Vitamin K antagonists.
